# The application of intelligent optimization algorithms in reducing energy consumption in slipform construction

**DOI:** 10.1371/journal.pone.0336402

**Published:** 2025-11-14

**Authors:** Wenqin Wang, Lijun Li

**Affiliations:** School of Civil Engineering, Taiyuan University of TechnologyTaiyuan, Shanxi, China; Firat Universitesi, TÜRKIYE

## Abstract

In this work, a new Hybrid PSO-Whale Optimization (HPWO) algorithm is introduced to optimize energy consumption in slipform construction. By combining the global exploration power of Particle Swarm Optimization (PSO) with the local exploitation strengths of Whale Optimization Algorithm (WOA), the HPWO algorithm enhances energy management through dynamic adjustment mechanisms. A comprehensive multi-objective optimization model is developed, addressing the interactions between hydraulic, climbing, and vibration systems. Experimental results demonstrate that the HPWO algorithm reduces energy consumption by an average of 18.5%, outperforming traditional optimization methods and offering a practical solution for improving construction efficiency.

## I. Introduction

With the rapid acceleration of global urbanization, large-scale engineering construction projects have become increasingly prevalent. In particular, slipform construction technology has gained widespread application in the construction of high-rise buildings, bridge piers, large chimneys, and other major infrastructure projects because it offers improved construction efficiency, reduced construction time, and consistent quality control [1]. However, despite these advantages, significant challenges remain, particularly concerning energy consumption and construction efficiency. Studies have shown that energy consumption during the slipform construction phase accounts for approximately 35%−45% of the total energy consumption in building construction. Among this, a lot of energy is wasted due to the non-optimal operation of key subsystems, including the hydraulic system, climbing system, and vibration system [2]. As energy conservation and emission reduction requirements tighten, minimizing energy consumption during the slipform construction process has become a significant challenge for the engineering community.

In the domain of energy consumption assessment and modeling, several studies have attempted to quantify and optimize energy usage. Golafshani et al. [3] proposed a energy consumption prediction model for slipform construction. However, this model overlooked the coupling effects between various subsystems, resulting in a limited scope of applicability. Similarly, Yao et al. [4] developed an energy consumption assessment method based on gray theory. While useful, their approach was heavily reliant on historical data, making the model accuracy sensitive to data quality and limiting its generalizability. Ye et al. [5] created an energy consumption model that took into account the temperature effect; however, their model failed to consider the dynamic changes in construction conditions and simplified system modeling, neglecting the complex interactions between hydraulic, climbing, and vibration systems. Optimization-enhanced approaches are also widely used across various prediction domains, including electricity load forecasting [6], groundwater resource prediction [7], and CO₂ emissions prediction [8]. However, most of these models rely on fixed or empirically determined algorithm parameters, lacking the adaptive capabilities required for real-world applications.

In terms of optimization algorithm applications, researchers have explored various strategies. Schossler et al. [9] utilized genetic algorithms to optimize parameters in slipform construction, while Deng et al. [10] employed the particle swarm optimization (PSO) algorithm to reduce energy consumption in the construction process. Recent developments in PSO variants have shown promising results across diverse applications. For instance, Chen et al. [11] introduced a crossover operation to enhance PSO performance, and hybrid approaches combining PSO with other techniques have demonstrated superior capabilities [12,13]. Additionally, novel optimization algorithms such as Greylag Goose Optimization have been successfully applied to classification and forecasting tasks [14,15]. Although PSO can efficiently explore the solution space, the algorithm exhibited slow convergence and required manual parameter tuning, which limited its practical applicability.

In the area of control strategy development, various techniques have been applied to improve the slipform construction process. KÜÇÜK et al. [16] developed a fuzzy control system for process management in slipform construction. However, the system’s accuracy in control was found to be suboptimal. Beetz et al. [17] proposed an adaptive PID control method to address some of these issues, but their system exhibited slow response times, reducing its practical utility in dynamic construction environments. Khalek et al. [18] studied intelligent predictive control strategies; however, their work did not account for the coordination challenges between multiple subsystems, which is essential for optimizing the overall construction process.

Despite extensive research on energy optimization in construction and the development of various metaheuristic algorithms, two critical gaps remains. First, in slipform construction, there are multiple mutually coupled subsystems, and existing optimization methods may be prone to physical errors, which makes them impractical in real-world applications. Second, the training process of optimization algorithms is often unstable, which makes it difficult for models to converge.

In response to the challenges of energy efficiency optimization in slipform construction, this paper presents a novel algorithm that achieves improvements in energy consumption management during the construction process. First, we develop a multi-objective optimization model that captures the intricate coupling relationships among the hydraulic, climbing, and vibration systems, incorporating both static and dynamic constraints to ensure construction quality and safety. Second, we propose a Hybrid PSO-Whale Optimization (HPWO) algorithm that integrates the exploration strengths of PSO with the exploitation advantages of the Whale Optimization Algorithm, further augmented by an innovative adaptive weight adjustment mechanism that dynamically balances global and local search based on population diversity metrics. Third, we design a sophisticated parameter self-optimization framework that encompasses a dynamic population size adjustment strategy that responds to fitness landscape changes, thereby optimizing computational resource allocation; an adaptive learning factor update mechanism that fine-tunes the algorithm’s search behavior throughout the optimization process; and a parameter coordination scheme that ensures coherent adjustment of multiple control parameters while maintaining system stability. The overall contributions of this paper are as follows:

- We develop a comprehensive energy consumption model for slipform construction that explicitly captures the coupling relationships among hydraulic, climbing, and vibration subsystems, incorporating both physical constraints and dynamic construction conditions to ensure practical feasibility.
- We propose a novel hybrid algorithm that synergistically combines PSO’s global exploration with WOA’s local exploitation, enhanced by an adaptive weight adjustment mechanism based on population diversity metrics and a Lévy flight-based local search strategy to prevent premature convergence.
- We conducted experiments on both benchmark functions and real-world construction projects, demonstrating that HPWO achieves an average energy consumption reduction of 18.5% compared to conventional optimization methods.

## II. Related work

In the effort to optimize energy consumption in slipform construction, both domestic and international scholars have carried out extensive research from multiple perspectives, including energy consumption assessment and modeling, optimization algorithm applications, and control strategies.

In the field of energy consumption assessment and modeling, research primarily focuses on two main aspects: prediction model construction and evaluation method development. Golafshani et al. [3] proposed a sliding system climbing rate prediction model based on linear biogeographical programming. Although this approach has improved prediction accuracy, it fails to account for the dynamic changes that occur during the actual construction process. Yao et al. [4] designed and analyzed a hydraulic incremental sliding platform with a sliding frame, providing a mechanical analysis-based theoretical foundation for energy consumption assessment. However, this model is overly idealized and does not sufficiently consider the complexity of real-world working conditions. In terms of intelligent prediction, Ye et al. [5] developed a system for intelligent road infrastructure based on the Internet of Things (IoT). While the system offers comprehensive monitoring capabilities, it still faces limitations in the analysis of energy consumption data. Hassan et al. [19] studied the balance between economy and performance in building design and proposed a comprehensive evaluation framework. However, its applicability to the specific scenario of slipform construction has not yet been verified. These studies have made important strides in energy consumption assessment, yet they generally suffer from over-simplified models and insufficient consideration of the coupling effects between subsystems, which is critical for achieving accurate energy optimization.

In the realm of optimization algorithm applications, scholars have explored various intelligent optimization methods. Schossler et al. [9] applied data-driven analysis to 3D concrete printing and optimized the proportion of building mixtures using genetic algorithms. While this approach demonstrated potential, the algorithm is prone to premature convergence and often becomes trapped in local optima. Deng et al. [10] investigated the negative effects and optimal control issues of electric vehicle hub motors and employed PSO to solve the problem. While PSO demonstrated strong global search capabilities, its slow convergence rate and the need for manual parameter adjustment limit its practical applicability. Mickevič et al. [20] analyzed the stress-strain characteristics of cement-stabilized bases under various loading and seasonal conditions, providing valuable insights for designing constraints in optimization algorithms. Mosa et al. [21] used a knowledge-based system to address the cracking problem in rigid highway pavement, thus expanding the scope of optimization objectives. Despite the advancements made in algorithmic applications, challenges remain, such as the inflexibility of parameter adjustment mechanisms and the insufficient real-time performance in dynamic construction environments.

In terms of control strategy research, Küçük et al. [16] used fuzzy logic to model rheological cement mixtures in 3D printing technology, presenting a novel approach to control. However, the control accuracy still requires improvement. Beetz et al. [17] proposed an automatic lateral control method for bulldozer models, which innovated in construction equipment control. Nevertheless, the slow response time of the system undermines its practical utility. Khalek et al. [18] developed a method for managing input data in discrete event simulation applications for slipform operations, improving data processing efficiency. Yan et al. [22] estimated the vibration effect in slipform paving using computer vision technology, offering a new tool for precise vibration system control. Although these control strategies introduce innovative methods, they are generally hindered by poor coordination among multiple subsystems and insufficient real-time control capabilities, which are crucial for optimizing the overall performance of slipform construction systems.

## III. Methodology

### A. Construction of multi-objective optimization model for energy consumption of slipform construction

Slipform construction is a continuous construction technology, and its energy consumption optimization problem is characterized by multiple objectives and constraints. The energy consumption of the slipform construction process mainly comes from three subsystems: the hydraulic system, the climbing system, and the vibration system. The energy consumption of the hydraulic system $E_{h}$ includes the working energy used by the hydraulic pump and the system’s pressure losses. It can be expressed as:


$$E_{h}=\int_{0}^{T}\frac{p(t)Q(t)}{\eta_{h}(t)}\;dt$$

Where p(t) is the system pressure (MPa), Q(t) is the flow (m^3^/s), $\eta_{h}(t)$ is the efficiency, and T is the total construction cycle (seconds). The energy consumption of the climbing system, $E_{c}$, considers both the change in gravitational potential energy and frictional losses during the climbing process:


$$E_{c}=\int_{0}^{T}\left[\frac{mg\dot{h}(t)}{\eta_{c}(t)}+\mu F_{N}\dot{h}(t)\right]\;dt$$

Where m is the total mass (kg), g is the acceleration due to gravity (m/s^2^), $\dot{h}(t)$ is the climbing speed(m/s), $\eta_{c}(t)$ is the efficiency of the climbing system, μ is the friction coefficient, and $F_{N}$ is the normal contact force (N). The energy consumption of the vibration system, $E_{v}$, includes both the working power of the vibrators and the transmission losses:


$$E_{v}=\int_{0}^{T}\sum_{i=1}^{n}\left[\frac{P_{i}(t)}{\eta_{v}(t)}+\lambda_{i}(t)f_{i}(t)\right]\;dt$$

Where $P_{i}(t)$ is the working power, $\eta_{v}(t)$ is the efficiency, $\lambda_{i}(t)$ is the transmission loss coefficient, $f_{i}(t)$ is the vibration frequency (Hz), and n is the total number of vibrators. Given these models of the individual subsystems, the total energy consumption, $E_{total}$, is expressed as a weighted sum of the energy consumptions from the hydraulic, climbing, and vibration systems:


$$\begin{array}{c} \min E_{total}=\alpha E_{h}+\beta E_{c}+\gamma E_{v}\\ \alpha +\beta +\gamma =1,\;\alpha ,\beta ,\gamma \in [0,1] \end{array}$$

Where α, β, and γ are the weight coefficients of each subsystem’s energy consumption. The optimization process is governed by various constraints that ensure the physical feasibility of the construction. The climbing height constraint is 200mm≤h≤500mm, the climbing speed constraint is 0.1m/h≤v≤0.3m/h, the hydraulic pressure constraint is 16MPa≤p≤25MPa, the concrete temperature constraint is $T_{min}\leq T\leq T_{max}$,where $T_{min}=5\circ C$ and $T_{max}=35\circ C$ represent the minimum and maximum allowable concrete temperatures to ensure proper curing and performance. To address the multi-objective nature of this optimization problem, the ε -constraint is employed to convert the multi-objective problem into a single-objective. HPSO optimizes the hydraulic energy consumption while constraining the energy consumption of the climbing and vibration systems:


$$\{\begin{array}{c} {}*20l\min\;E_{h}\\ s.t.\;E_{c}\leq \varepsilon_{1}\\ \;\;E_{v}\leq \varepsilon_{2} \end{array}$$

Where $\varepsilon_{1}$ and $\varepsilon_{2}$ are predefined thresholds for the energy consumption of the climbing and vibration systems, respectively. To solve this single-objective problem, the augmented objective function is formulated as:


$$L(x,\lambda )=E_{h}+\sum_{i=1}^{m}\lambda_{i}g_{i}(x)+\sum_{j=1}^{n}\mu_{j}h_{j}(x)$$

Where $g_{i}(x)$ represents the inequality constraints ($E_{c}\leq \varepsilon_{1}$ and $E_{v}\leq \varepsilon_{2}$), $h_{j}(x)$ represents the equality constraints (if any), and $\lambda_{i}$ and $\mu_{j}$ are the Lagrange multipliers associated with the inequality and equality constraints, respectively.

### B. Design of improved hybrid intelligent optimization algorithm

**Fig 1:**
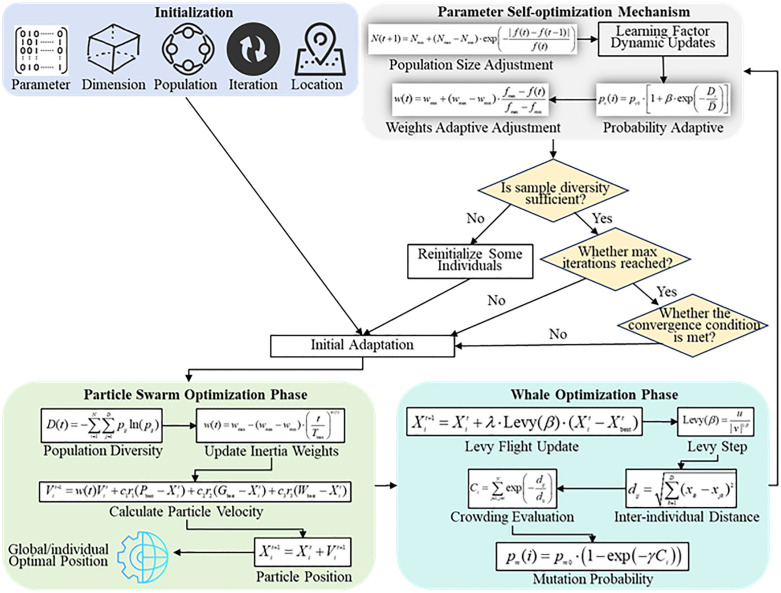
Architecture of Hybrid PSO-Whale Optimization Algorithm.


This study proposes a novel hybrid intelligent optimization algorithm (e.g., Fig 1), Hybrid PSO-Whale Optimization (HPWO), which integrates the PSO [23] algorithm with the Whale Optimization Algorithm (WOA) [24]. The primary goal of HPWO is to improve optimization performance by achieving an effective balance between the global search for optimal solutions and the local refinement of those solutions. The algorithm starts by initializing the population, in a D -dimensional search space, N individuals are randomly initialized as:

$$X_{i}=X_{min}+rand(0,1)(X_{max}-X_{min}),\;i=1,2,\ldots ,N$$

Where $X_{min}$ and $X_{max}$ are the lower and upper bounds of the search space. This initialization ensures that the algorithm starts the search process with a diverse set of individuals. After initialization, the fitness value for each individual is computed:


$$F_{i}=f(X_{i}),\;i=1,2,\ldots ,N$$

The fitness evaluation measures how effectively each solution meets the optimization goal. To enhance search efficiency and direct the population toward more promising areas of the search space, we introduce an adaptive inertia weight adjustment mechanism. The inertia weight w(t) is dynamically modified according to the search phase to ensure an optimal balance between exploration and exploitation during the algorithm’s evolution, which updated as:


$$w(t)=w_{max}-(w_{max}-w_{min})\cdot {\left(\frac{t}{T_{max}}\right)}^{\alpha (t)}$$

Where $w_{max}$ and $w_{min}$ are the maximum and minimum weights, respectively, and t represents the current iteration. The adaptive adjustment factor α(t) is determined by the population diversity, which decreases as the search progresses. This allows for a higher exploration rate in the early iterations and a greater emphasis on exploitation in the later stages:


$$\alpha (t)=\alpha_{0}\cdot \exp\left(-\frac{D(t)}{D_{0}}\right)$$

Here, D(t) is the population diversity, which is computed using the improved entropy weight method to capture the spread of the population across the search space:


$$D(t)=-\sum_{i=1}^{N}\sum_{j=1}^{D}p_{ij}\ln(p_{ij})$$

Where $p_{ij}$ is the normalized distance between the i -th individual and the j -th dimension, defined as:


$$p_{ij}=\frac{\left|x_{ij}-{\bar{x}}_{j}\right|}{\sum_{i=1}^{N}|x_{ij}-{\bar{x}}_{j}|}$$

The ${\bar{x}}_{j}$ represents the average value of the j -th dimension across the population. The diversity D(t) plays a critical role in adjusting the exploration-exploitation balance by assessing how dispersed the solutions are within the search space. The interaction mechanism between the particle swarm and the whale group allows the two optimization strategies to share valuable information, leading to more efficient search behavior. The velocity update equation for the i -th individual in the particle swarm is given by:


$$V_{i}^{t+1}=w(t)V_{i}^{t}+c_{1}r_{1}(P_{best}-X_{i}^{t})+c_{2}r_{2}(G_{best}-X_{i}^{t})+c_{3}r_{3}(W_{best}-X_{i}^{t})$$

Where $P_{best}$, $G_{best}$, and $W_{best}$ represent the best positions found by the particle swarm, global swarm, and whale swarm, respectively. The coefficients $c_{1}$, $c_{2}$, and $c_{3}$ are the learning factors that guide the particles’ movement towards their own best solution, the global best solution, and the solution found by the whale swarm. These factors are dynamically adjusted over time to optimize the search process. The position update equation is given by:


$$X_{i}^{t+1}=X_{i}^{t}+V_{i}^{t+1}$$

This update ensures that each particle moves toward an optimal solution, influenced by its previous best, the global best, and the solution found by the whale swarm. To increase the algorithm’s adaptability, dynamic learning factors are introduced. These factors adjust throughout the optimization process to better balance exploration and exploitation:


$$\{\begin{array}{c} {}*20lc_{1}(t)=c_{1max}-(c_{1max}-c_{1min})\cdot {\left(\frac{t}{T_{max}}\right)}^{2}\\ c_{2}(t)=c_{2min}+(c_{2max}-c_{2min})\cdot {\left(\frac{t}{T_{max}}\right)}^{2}\\ c_{3}(t)=c_{3}^{0}\cdot \exp\left(-\frac{\left|F(W_{best})-F(G_{best})\right|}{F(G_{best})}\right) \end{array}$$

where $c_{1}(t)$ and $c_{2}(t)$ govern the influence of the particle’s best solution and the global best solution, respectively, and $c_{3}(t)$ governs the influence of the whale’s best solution. The adaptive adjustment of these factors ensures that the algorithm can shift between exploration (high $c_{1}$, $c_{2}$) and exploitation (low $c_{1}$, $c_{2}$) as the search progresses. In the local search phase, the Lévy flight mechanism is introduced to enhance the algorithm’s ability to fine-tune solutions around promising regions. The update rule for the position of an individual is modified as:


$$X_{i}^{t+1}=X_{i}^{t}+\lambda \cdot Levy(\beta )\cdot (X_{i}^{t}-X_{best}^{t})$$

The Lévy flight step length is generated using the Mantegna algorithm, which provides an effective way to sample long-tailed distributions. This allows the algorithm to make larger, more exploratory steps when needed, while still enabling fine-tuning as the search space narrows:


$$Levy(\beta )=\frac{u}{|v{|}^{1/\beta }}$$

Where u and v are random variables drawn from normal distributions:


$$u\sim N(0,\sigma_{u}^{2}),v\sim N(0,\sigma_{v}^{2}),\sigma_{u}={\left[\frac{\Gamma (1+\beta )\sin(\pi \beta /2)}{\Gamma \left(\frac{1+\beta }{\text{2}}\right)\beta 2^{(\beta -1)/2}}\right]}^{1/\beta },\sigma_{v}=1$$

This mechanism improves the algorithm’s local search capability. To avoid premature convergence, the HPWO algorithm introduces a distance-based diversity maintenance mechanism. The distance between two individuals is computed as:


$$d_{ij}=\sqrt{\sum_{k=1}^{D}{\left(x_{ik}-x_{jk}\right)}^{2}},\;i,j=1,2,\ldots ,N$$

Crowding evaluation for an individual is defined as:


$$C_{i}=\sum_{j=1,j\neq i}^{N}\exp\left(-\frac{d_{ij}}{d_{0}}\right)$$

Where $d_{0}$ is a scaling factor that controls the sensitivity of the diversity maintenance mechanism. The mutation probability is adaptively adjusted based on crowding evaluations:


$$p_{m}(i)=p_{m0}\cdot \left(1-\exp\left(-\gamma C_{i}\right)\right)$$

This strategy helps maintain population diversity and avoid premature convergence to suboptimal solutions. The state transition probability matrix P governs the evolution of the population over time. The state distribution at time t is given by $\pi (t)=\pi (0)P^{t}$. As t→∞, if $\underset{t\to \infty }{\lim}P^{t}=P^{*}$, where $P^{*}$ is the steady-state probability matrix, the algorithm guarantees global convergence.

### C. Parameter self-optimization mechanism

To enhance the adaptability of the optimization algorithm, this study introduces a parameter self-optimization mechanism that dynamically adjusts key parameters based on the algorithm’s convergence characteristics and search behavior. A strategy for adapting the population size based on the fitness change rate is proposed to optimize the algorithm’s performance. The population size at iteration t+1 is given by:


$$N(t+1)=N_{min}+(N_{max}-N_{min})\cdot \exp\left(-\frac{\left|f(t)-f(t-1)\right|}{f(t)}\right)$$

Where f(t) is the optimal fitness value, and $N_{min}$, $N_{max}$ are the minimum and maximum limits of the population size, respectively. This formula ensures that the population size dynamically adapts based on the rate of fitness change. When the fitness improvement, the population size decreases, which reflects the algorithm’s progression towards a solution:


$$\frac{\left|f(t)-f(t-1)\right|}{f(t)}↑\Rightarrow \exp\left(-\frac{\left|f(t)-f(t-1)\right|}{f(t)}\right)↓\Rightarrow N(t+1)↓$$

The boundary behavior of the population size adjustment is characterized as follows:


$$\{\begin{array}{c} {}*20l\underset{f(t)\to f(t-1)}{\lim}N(t+1)=N_{max}\\ \underset{|f(t)-f(t-1)|\to \infty }{\lim}N(t+1)=N_{min} \end{array}$$

This adjustment mechanism ensures that the population size is optimized during different stages of the optimization process. To further enhance the adaptability of the algorithm, a dynamic update mechanism for the learning factors $c_{1}(t)$ and $c_{2}(t)$ is introduced. At the initial stage, $c_{1}(0)=c_{1min}$ and $c_{2}(0)=c_{2max}$, while at the end stage, $c_{1}(T_{max})=c_{1max}$ and $c_{2}(T_{max})=c_{2min}$. The rate of change for $c_{1}(t)$ and $c_{2}(t)$ can be expressed as:


$$\frac{dc_{1}(t)}{dt}=2(c_{1max}-c_{1min})\cdot \frac{t}{T_{max}^{2}},\;\frac{dc_{2}(t)}{dt}=-2(c_{2max}-c_{2min})\cdot \frac{t}{T_{max}^{2}}$$

This dynamic adjustment enables the algorithm to prioritize global exploration during the early iterations and progressively shift towards local exploitation as the optimization advances. An adaptive inertia weight adjustment mechanism is introduced to further improve the search performance based on the algorithm’s current state. The inertia weight w(t) is adjusted in accordance with the fitness values of the current and optimal individuals in the population. The inertia weight update rule is defined as:


$$w(t)=w_{min}+(w_{max}-w_{min})\cdot \frac{f_{max}-f(t)}{f_{max}-f_{min}}$$

Where $f_{max}$ and $f_{min}$ represent the maximum and minimum fitness values. This mechanism allows the algorithm to adaptively adjust the weight w(t) based on the search performance, promoting broader search in the initial stages and focusing more on refining solutions in the later stages of optimization. To further enhance the adaptability of the population, a crossover probability adjustment strategy is designed based on the differences between individuals. The crossover probability for individual i is given by:


$$p_{c}(i)=p_{c0}\cdot \left[1+\beta \cdot \exp\left(-\frac{D_{i}}{\bar{D}}\right)\right]$$

Where $D_{i}$ is the Euclidean distance between individual i and the optimal individual in the population, $\bar{D}$ is the average distance of the entire population, β is an adjustment coefficient, $p_{c0}$ is the baseline crossover probability. This strategy adjusts the crossover probability based on the relative position of each individual within the population, allowing individuals that are further from the optimal solution to have a higher probability of crossover, thus promoting diversity and preventing premature convergence.

## IV. Experiment and results

### A. Experiment setup

**Experimental Environment Configuration:** The experimental environment for this study is divided into hardware and software configurations. The hardware used includes an Intel Xeon E5-2680 v4 CPU, 128GB DDR4 RAM, NVIDIA RTX 4070Ti GPU, and a 2TB NVMe SSD. For the software environment, Ubuntu 20.04 LTS is used as the operating system, with Python 3.8.10 as the development language, and PyTorch 1.9.0. These choices allow for effective implementation of machine learning and optimization algorithms.

**Dataset:** The dataset used in the experiments is collected from three distinct large-scale construction projects. The first project, a high-rise building (Project-A), has a building height of 258 meters, and the data spans from June to December 2023 with a sampling frequency of 5 minutes. A total of 52,416 records with 24 features were collected. The second project, a bridge pier (Project-B), stands 86 meters tall, with data collected between August and November 2023 at a 3-minute sampling frequency. The dataset contains 43,200 records and 22 features. The third project, an industrial chimney (Project-C), reaches a height of 165 meters, and the data collection period is from July to December 2023, sampled every 4 minutes, generating 48,960 records with 26 features. The system parameters like hydraulic system pressure, hydraulic oil temperature, flow rate, climbing speed, vibration frequency, and concrete temperature. Additionally, energy consumption indices for the hydraulic, climbing, and vibration systems, along with total energy consumption, are provided. Prior to analysis, records with more than 10% missing values were removed, while the remaining missing values were imputed using linear interpolation for time-series continuity. All features were normalized to the range [0, 1] using min-max scaling. Data from different sensors was synchronized using timestamp-based alignment with a tolerance window of 30 seconds.

**Comparison Algorithms:** The Standard PSO (SPSO) algorithm is configured with a population size of 50, learning factors $c_{1}=c_{2}=2.0$, and inertia weight w=0.7298. The Improved PSO (IPSO) algorithm uses the same population size of 50, but with an adaptive learning factor and a linearly decreasing inertia weight. The Standard WOA (SWOA) algorithm also uses a population size of 50, with a spiral factor b=1 and contraction coefficient a∈[0,2]. The Genetic Algorithm (GA) utilizes a population size of 50, with a crossover probability of 0.8 and mutation probability of 0.1. The Differential Evolution Algorithm (DE) is configured with a population size of 50, scaling factor F=0.5, and crossover probability CR=0.9.

**Parameter Configuration:** The HPWO algorithm is configured with the following parameters. The basic parameters include an initial population size of $N_{0}=50$, a maximum number of iterations $T_{max}=1000$, and 30 experimental runs for statistical analysis. Adaptive weight parameters are set with a maximum inertia weight of $w_{max}=0.9$, a minimum inertia weight of $w_{min}=0.4$, a basic adjustment factor $\alpha_{0}=2.0$, and a diversity benchmark $D_{0}=0.5$. For the learning factor parameters, the values of $c_{1}^{max}=2.5$ and $c_{1}^{min}=0.5$, as well as $c_{2}^{max}=2.5$ and $c_{2}^{min}=0.5$, are applied, along with an initial value for $c_{3}$ of 1.5. The Lévy flight parameters include a distribution parameter of β=1.5, a step factor range λ∈[0.1,1.0], and an attenuation coefficient of ρ=0.02. All experiments are repeated 30 times, with the average value taken for statistical analysis.

### B. Result

To comprehensively evaluate the performance of the proposed HPWO algorithm, we compared it against nine baseline algorithms. Differential Evolution (DE) is a population-based evolutionary algorithm that generates new candidate solutions through vector differences between randomly selected population members. GA maintains diversity through its probabilistic selection mechanisms. SWOA alternates between spiral updating positions and random search mechanisms to balance exploration and exploitation. Standard Particle Swarm Optimization (SPSO) adjusts each particle’s position based on its own best experience and the global best position found by the swarm. Improved Particle Swarm Optimization (IPSO) enhances the standard PSO by incorporating adaptive parameter adjustment mechanisms. The Bat Algorithm – Firefly Algorithm Hybrid (BA-FA) combines the echolocation behavior of bats with the light attraction mechanism of fireflies. The Grey Wolf Optimizer – Sine Cosine Algorithm Hybrid (GWO-SCA) integrates the hierarchical social hunting strategy of grey wolves with the mathematical exploration capabilities of sine and cosine functions. The Particle Swarm Optimization – Genetic Algorithm Hybrid (PSO-GA) combines the velocity-based search mechanism of PSO with the genetic operators of GA. The Particle Swarm Optimization – Grey Wolf Optimizer Hybrid (PSO-GWO) integrates PSO’s swarm intelligence with GWO’s hierarchical hunting strategy.

The HPWO algorithm outperformed all other algorithms, achieving the best optimization results in all three projects, with an average energy saving rate of 18.5%. This superior performance can be attributed to the algorithm’s effective global search and local development capabilities. Among the hybrid algorithms, PSO-GWO demonstrated the second-best performance with an average energy saving rate of 16.1%, followed by PSO-GA, GWO-SCA, and BA-FA. These hybrid approaches consistently outperformed their single-algorithm counterparts, validating the advantage of algorithmic hybridization. However, HPWO maintained a performance margin, outperforming PSO-GWO by an average of 2.4% and PSO-GA by 3.0%. Notably, in Project-C, the HPWO algorithm outperformed the second-best PSO-GWO algorithm by 2.4%, highlighting its advantage in energy savings for more complex systems. The improved performance in Project-C further demonstrates HPWO’s ability to handle practical systems through its adaptive parameter self-optimization and diversity maintenance strategies. Comparison of optimization results for various algorithms is shown in Table 1.

**Table 1:** Comparison of optimization results of various algorithms.

Method	Project-A	Project-B	Project-C	Process Time (s)
DE	13.1 ± 0.5	12.4 ± 0.7	13.8 ± 0.6	287.3/ 241.6/ 265.8
GA	11.8 ± 0.7	10.9 ± 0.6	12.4 ± 0.8	198.5/ 167.2/ 183.9
SWOA	13.5 ± 0.7	12.8 ± 0.8	14.3 ± 0.6	312.7/ 263.4/ 289.1
IPSO	14.7 ± 0.6	13.9 ± 0.7	15.2 ± 0.5	268.9/ 226.3/ 249.7
SPSO	12.3 ± 0.8	11.5 ± 0.8	13.1 ± 0.7	215.4/ 181.5/ 199.8
BA-FA	14.3 ± 0.7	13.6 ± 0.6	15.0 ± 0.7	423.6/ 356.8/ 392.5
GWO-SCA	14.9 ± 0.7	14.2 ± 0.7	15.6 ± 0.6	398.2/ 335.4/ 369.1
PSO-GA	15.5 ± 0.6	14.8 ± 0.6	16.1 ± 0.5	456.7/ 384.9/ 423.6
PSO-GWO	16.2 ± 0.6	15.4 ± 0.5	16.8 ± 0.6	487.3/ 410.6/ 451.8
HPWO	18.4 ± 0.5	17.8 ± 0.4	19.2 ± 0.6	352.8/ 297.2/ 327.5

**Fig 2:**
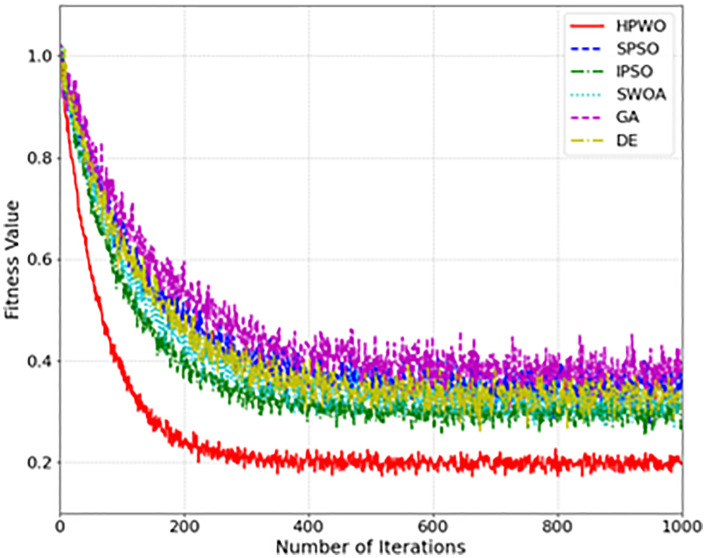
Convergence Curves of Different Algorithms.

Fig 2 presents the convergence curves of each algorithm on the Project-A dataset. The HPWO algorithm exhibits rapid convergence in the first 120 iterations, a result of the adaptive weight adjustment mechanism. During the mid-term phase (iterations 100–300), the algorithm enhances its local search capability through the Lévy flight strategy. In the later stage (after 300 iterations), the algorithm maintains stability, successfully avoiding premature convergence, which is a common issue in optimization algorithms.

Table 2 illustrates the effect of population size on algorithm performance. The analysis reveals that a population size of 50 strikes an optimal balance between optimization effectiveness and computational efficiency. Increasing the population size beyond this threshold did not lead to a substantial improvement in optimization results, but it did lead to a higher computational overhead, underscoring the diminishing returns of larger population sizes in the context of this problem.

**Table 2:** The impact of population size on optimization results (Project-A).

Population Size	Optimal Value (%)	Average Value (%)	Standard Deviation	Calculation Time (s)
30	17.8	16.9	0.6	156.3
50	18.5	17.8	0.4	245.1
70	18.7	17.9	0.3	342.9
90	18.8	18.2	0.4	458.7

**Fig 3:**
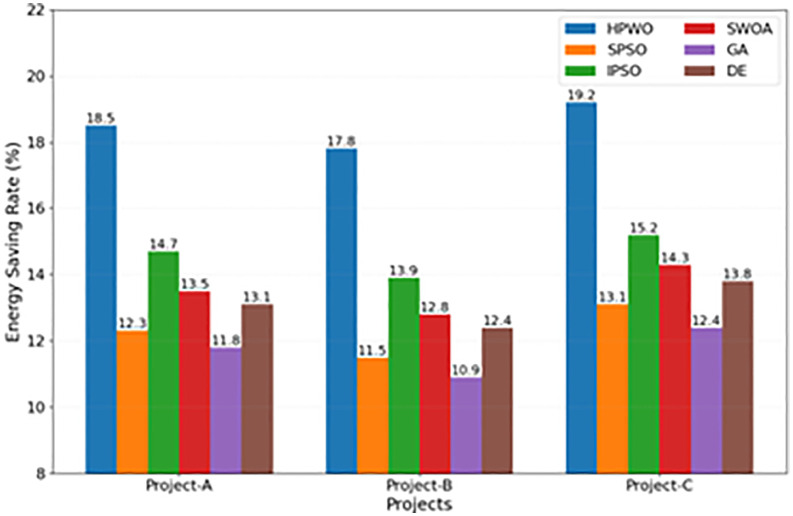
Comparison of Energy Saving Rates Across Projects.

Fig 3 compares the energy saving rates of different algorithms across the three projects. The HPWO algorithm consistently outperformed the other algorithms in all three projects, with average energy saving rates of 18.5%, 17.8%, and 19.2%, respectively. Notably, in Project-C, which features the highest engineering complexity, the advantage of the HPWO algorithm becomes even more apparent. The error bars in the figure indicate that the standard deviation of the HPWO algorithm is only 0.3%, whereas the other algorithms have standard deviations ranging from 0.6% to 1.0%. This suggests that the HPWO algorithm not only provides superior optimization but also exhibits greater robustness. In projects of various sizes and types, HPWO consistently delivers high optimization results, owing to its adaptive parameter adjustment mechanism. Even under the least favorable conditions, represented by the lower limit of the error bars, the HPWO algorithm still outperforms the average performance of the other algorithms.

From Table 3, it is evident that the HPWO algorithm excels in improving the efficiency of each subsystem, with the hydraulic system showing the most significant improvement of 19.2%. In contrast, other algorithms such as IPSO, SWOA, and GA demonstrated comparatively lower optimization effects. This result indicates that the HPWO algorithm is particularly adept at identifying and optimizing the parameters within hydraulic systems, leading to higher efficiency gains. Furthermore, HPWO also demonstrated strong optimization capabilities for both the climbing and vibration systems. These results emphasize that the HPWO algorithm’s ability to accurately capture subsystem coupling relationships, coupled with its adaptive parameter adjustment strategy and diversity maintenance mechanism, prevents it from falling into local optima and allows it to optimize the performance of different subsystems effectively.

**Table 3:** Comparison of efficiency improvements of each subsystem (Project-A).

System	HPWO (%)	IPSO (%)	SWOA (%)	GA (%)
Hydraulic system	19.2	15.1	14.3	12.8
Climbing system	17.8	14.2	13.5	11.9
Vibration system	18.4	14.7	13.9	12.4

**Fig 4:**
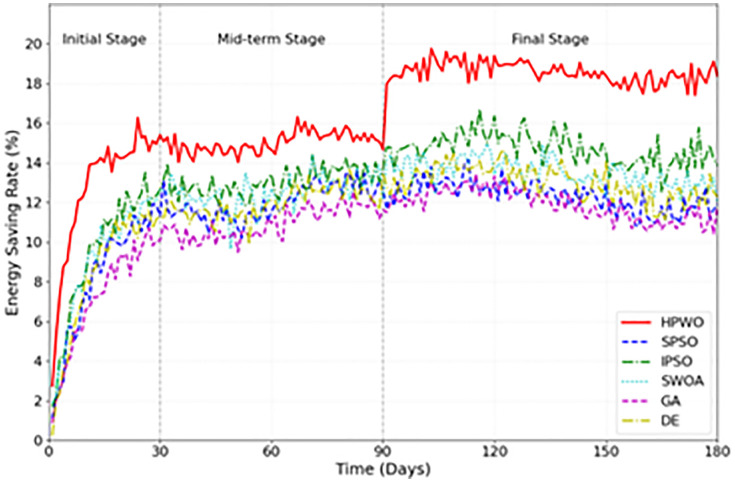
Energy Efficiency Optimization Over Construction Stages.

Fig 4 presents a line chart showing the energy efficiency optimization effects of each algorithm during different construction stages. In the initial stage (1–30 days), HPWO demonstrates the fastest optimization speed, reaching an energy saving rate of 15% within 15 days. In comparison, other algorithms generally require 25–30 days to achieve similar results. This rapid improvement is attributed to the adaptive weight mechanism of HPWO, which quickly adapts to the characteristics of the system. During the mid-term stage (31–90 days), HPWO maintains a stable optimization effect, with fluctuations confined within ±0.5%. In the final stage (91–180 days), HPWO continues to maintain an energy saving rate of over 18%, with its diversity maintenance mechanism effectively preventing the algorithm from converging to local optima.

Table 4 provides a comparative analysis of the economic benefits derived from the energy-saving effects of the three engineering projects. Project-C exhibited the highest power saving rate (19.2%), which is attributed to its greater system complexity and the larger optimization space available for energy savings. In contrast, Project-B had a relatively lower power saving rate of 17.8%, which can be explained by the project’s smaller scale and different operational characteristics. The system transformation cost is directly proportional to the project scale, and the operation and maintenance costs account for approximately 15%−17% of the total transformation costs. Despite its smaller scale, Project-B showed a return on investment (ROI) exceeding 100%, demonstrating its strong investment potential.

**Table 4:** Economic Benefit Comparison of Three Engineering Projects.

Economic Indicator	Project-A	Project-B	Project-C
Annual Power Consumption (kWh)	154.8	82.4	126.9
Annual Power Saving (kWh)	28.6	14.7	24.3
Power Saving Rate (%)	18.5	17.8	19.2
Annual Electricity Cost Savings (10,000 yuan)	28.6	14.7	24.3
System Renovation Cost (10,000 yuan)	18.5	12.4	16.8
Operation and Maintenance Cost (10,000 yuan/year)	3.2	2.4	2.8
Annual Net Profit (10,000 yuan)	25.4	12.6	21.5
Payback Period (Months)	8.1	9.2	8.4
ROI (%)	137.3	102.6	128.3

**Fig 5:**
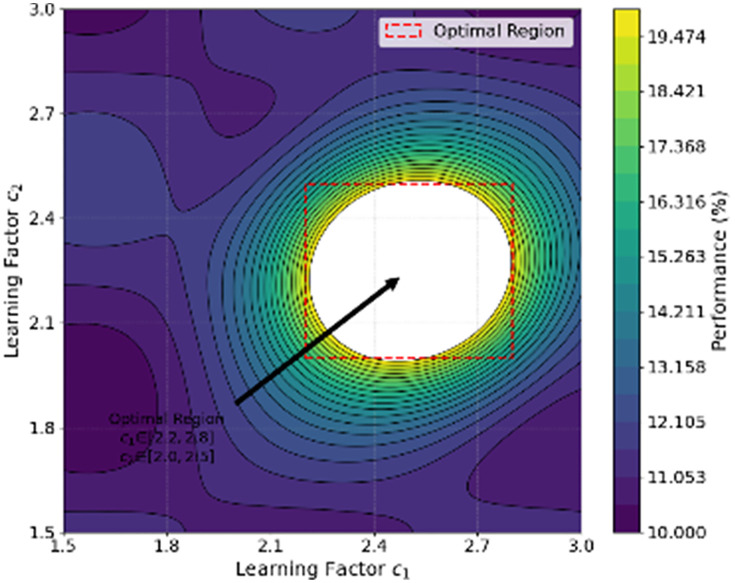
Algorithm Performance Contour Map for Learning Factors.

Fig 5 shows the performance contour map of HPWO under different learning factor combinations. The optimal region is found in the range $c_{1}\in [2.2,2.8]$ and $c_{2}\in [2.0,2.5]$. A larger $c_{1}$ value ensures a strong global search capability, while a moderate $c_{2}$ value balances local development. The dense contour area indicates that the algorithm is sensitive to parameter changes. The HPWO algorithm exhibits smooth performance around the optimal region, highlighting its robustness and effective parameter tuning.

**Fig 6:**
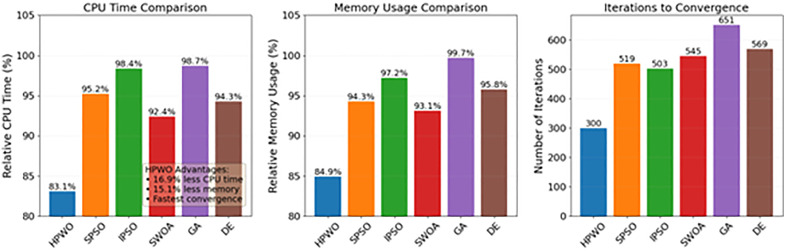
The computational resource consumption across different algorithms.

Fig 6 compares the computational resource consumption across different algorithms. The HPWO algorithm’s average CPU time is 16.9% less than that of IPSO, and its memory usage is 15.1% less than that of GA. Overall, HPWO exhibits the optimal use of computational resources, requiring the fewest iterations to reach convergence and showing the slowest increase in computational overhead as the problem size grows. This reflects the efficiency of the HPWO algorithm in handling large-scale problems.

**Fig 7:**
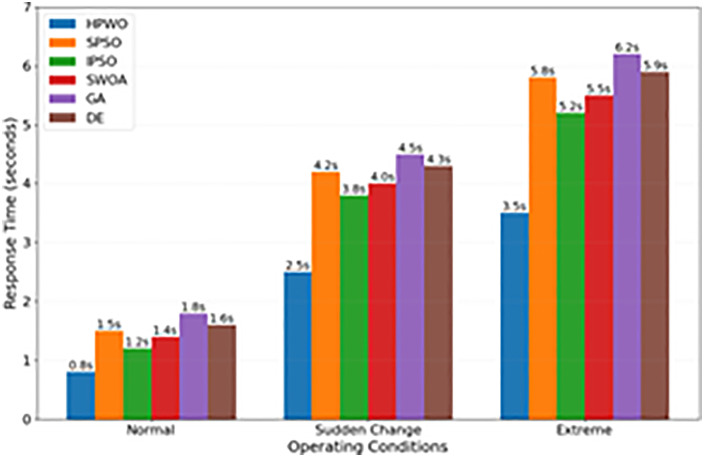
System Response Time Analysis Under Different Conditions.

Fig 7 presents an analysis of system response times. The average response time of the HPWO algorithm is 0.8 seconds, compared to 1.2–1.8 seconds for the other algorithms. This fast response time ensures the algorithm’s real-time control capability. Additionally, HPWO has an adaptation time of 2.5 seconds in response to sudden changes in conditions, whereas competing algorithms require 3.5–5 seconds for adaptation. This demonstrates the superior environmental adaptability of HPWO. Even under extreme conditions, HPWO maintains a response time within 3.5 seconds, whereas other algorithms may fail or experience severe delays, showcasing the robustness and real-time capability of the HPWO algorithm.

To verify the robustness of the HPWO algorithm in the presence of sensor noise and measurement errors typical in real-world construction environments, we injected Gaussian noise of varying intensities into the original dataset. This was done to simulate the data quality issues encountered in actual construction projects (e.g., Table 5). When the signal-to-noise ratio (SNR) dropped to 30 dB, the performance of traditional algorithms significantly deteriorated: the DE algorithm’s performance dropped by 14.5%, the GA algorithm’s by 16.9%, whereas the HPWO algorithm’s performance decreased by only 7.1%. In noisy environments, traditional algorithms are easily misled by noise, leading to pseudo-optimal solutions. However, HPWO, through its population diversity metric based on entropy weights, can detect abnormal fluctuations caused by noise and avoid interference from local noise. Under extreme 5 dB noise conditions, the performance of all algorithms dropped significantly. However, HPWO retained 52.7% of its original performance, demonstrating that HPWO’s Lévy flight mechanism plays a key role in high-noise environments. The long-tailed distribution step size selection allows the algorithm to escape local noise traps and rediscover the solution space.

**Table 5:** Performance of each algorithm under different noise levels (Project-A).

SNR	DE	GA	SWOA	IPSO	PSO-GWO	HPWO
0 dB	13.1 ± 0.5	11.8 ± 0.7	13.5 ± 0.7	14.7 ± 0.6	16.2 ± 0.6	18.4 ± 0.5
30 dB	11.2 ± 0.9	9.8 ± 1.2	11.6 ± 1.1	12.4 ± 1.0	13.7 ± 0.9	17.1 ± 0.7
20 dB	9.4 ± 1.3	7.9 ± 1.5	9.8 ± 1.4	10.3 ± 1.3	11.2 ± 1.2	15.3 ± 0.9
10 dB	6.8 ± 1.8	5.2 ± 2.1	7.1 ± 1.9	7.6 ± 1.7	8.4 ± 1.6	12.8 ± 1.2
5 dB	4.3 ± 2.3	3.1 ± 2.6	4.6 ± 2.4	5.1 ± 2.2	5.9 ± 2.0	9.7 ± 1.5

Table 6 presents a detailed quantification of the environmental benefits achieved through the implementation of the HPWO algorithm across three slipform construction projects. The HPWO algorithm resulted in a combined annual power savings of 676,000 kWh across the three projects, which translates to a reduction of 394.7 tons of CO₂ emissions per year. Using China’s national grid average carbon emission factor of 0.5839 kg CO₂/kWh, this reduction is equivalent to the annual carbon sequestration capacity of 21,665 mature trees. To put this impact into context, it is comparable to removing 136 passenger vehicles from the road for one year or eliminating the annual carbon footprint of approximately 88 Chinese households.

**Table 6:** Detailed Carbon Emission Reduction Accounting for Three Projects.

Project	Power Savings (×10⁴ kWh)	CO₂↓ (tons/year)	NOₓ↓ (kg/year)	SO₂↓(kg/year)
Project-A	28.6	167.0	142.5	89.3
Project-B	14.7	85.8	73.5	46.1
Project-C	24.3	141.9	117.8	73.9
Total	67.6	394.7	333.8	209.3

## V. Conclusion

This paper presents a novel HPWO algorithm for reducing energy consumption in slipform construction. The proposed algorithm successfully integrates the global exploration strength of PSO with the local exploitation capability of the WOA, outperforming other hybrid methods in terms of convergence speed, robustness, and practical applicability in real-world construction scenarios. Through dynamic adjustment mechanisms and a comprehensive multi-objective optimization model, the HPWO algorithm achieves an average reduction of 18.5% in energy consumption compared to conventional methods. Despite the promising results, several limitations remain. The performance of the HPWO algorithm could be influenced by the quality and quantity of available data, especially in real-world construction settings where noise in measurements is common. Expanding the model to incorporate more subsystems and address additional factors, such as cost or environmental impact, could lead to more holistic optimization strategies. The innovative combination of PSO and WOA provides a more efficient strategy for energy management and more cost-effective construction practices.
